# Isha Yoga Practices and Participation in Samyama Program are Associated with Reduced HbA1C and Systemic Inflammation, Improved Lipid Profile, and Short-Term and Sustained Improvement in Mental Health: A Prospective Observational Study of Meditators

**DOI:** 10.3389/fpsyg.2021.659667

**Published:** 2021-05-19

**Authors:** Senthilkumar Sadhasivam, Suresh Alankar, Raj Maturi, Amy Williams, Ramana V. Vishnubhotla, Sepideh Hariri, Mayur Mudigonda, Dhanashri Pawale, Sangeeth Dubbireddi, Senthil Packiasabapathy, Peter Castelluccio, Chithra Ram, Janelle Renschler, Tracy Chang, Balachundhar Subramaniam

**Affiliations:** ^1^Department of Anesthesia, Indiana University School of Medicine, Indianapolis, IN, United States; ^2^Vascular Surgery, Honorary Staff, University of Louisville, Louisville, KY, United States; ^3^Department of Ophthalmology, Eugene and Marilyn Glick Eye Institute, Indiana University School of Medicine, Indianapolis, IN, United States; ^4^Department of Psychiatry, Riley Hospital for Children, Indiana University School of Medicine, Indianapolis, IN, United States; ^5^Department of Radiology, Indiana University School of Medicine, Indianapolis, IN, United States; ^6^Department of Anesthesia, Critical Care and Pain Medicine, Sadhguru Center for a Conscious Planet, Beth Israel Deaconess Medical Center, Boston, MA, United States; ^7^Lawrence Berkeley National Laboratory, Berkeley, CA, United States; ^8^Cardiac Intensivist, Ascension Health, St. Vincent Hospital, Indianapolis, IN, United States; ^9^Department of Biostatistics, Indiana University School of Medicine, Indianapolis, IN, United States; ^10^Department of Radiology, University of Louisville Hospital, Louisville, KY, United States; ^11^Department of Labor Studies and Employment Relations, School of Management and Labor Relations, Rutgers University, New Brunswick, NJ, United States

**Keywords:** meditation, depression, anxiety, biomarkers, Isha, Samyama, triglycerides, mindfulness

## Abstract

**Background:** Meditation is gaining recognition as a tool to impact health and well-being. Samyama is an 8-day intensive residential meditation experience conducted by Isha Foundation requiring several months of extensive preparation and vegan diet. The health effects of Samyama have not been previously studied. The objective was to assess physical and emotional well-being before and after Samyama participation by evaluating psychological surveys and objective health biomarkers.

**Methods:** This was an observational study of 632 adults before and after the Isha Samyama retreat. All participants were invited to complete surveys. Controls included household significant others. Surveys were completed at baseline (T1), just before Samyama (T2), immediately after Samyama (T3), and 3 months later (T4) to assess anxiety, depression, mindfulness, joy, vitality, and resilience through validated psychometric scales. Voluntary blood sampling for biomarker analysis was done to assess hemoglobin (Hb), HbA1c, lipid profile, and C-reactive protein (CRP). Primary outcomes were changes in psychometric scores, body weight, and blood biomarkers.

**Results:** Depression and anxiety scores decreased from T1 to T3, with the effect most pronounced in participants with baseline depression or anxiety. Scores at T4 remained below baseline for those with pre-existing depression or anxiety. Vitality, resilience, joy, and mindfulness increased from T1 to T3 (sustained at T4). Body weight decreased by 3% from T1 to T3. Triglycerides (TG) were lower from T2 to T3. Participants had lower HbA1c and HDL at T2, and lower CRP at all timepoints compared with controls.

**Conclusions:** Participation in the Isha Samyama program led to multiple benefits. The 2-month preparation reduced anxiety, and participants maintained lower anxiety levels at 3 months post-retreat. Physical health improved over the course of the program as evidenced by weight loss and improved HbA1C and lipid profile. Practices associated with the Samyama preparation phase and the retreat may serve as an effective way to improve physical and mental health. Future studies may examine their use as an alternative therapy in patients with depression and/or anxiety.

**Clinical Trial Registration:** www.ClinicalTrials.gov, Identifier: 1801728792. Registered retrospectively on 4/17/2020.

## Background

Meditation is gaining credibility as a simple and effective tool to impact health and well-being. Researchers from a variety of disciplines have begun to investigate meditation-related cognitive, psychological, and physiological changes (Brandmeyer et al., 2019). As meditation-related research has grown exponentially since the 1990's, there are now over 5,000 peer-reviewed publications from the National Library of Medicine (pubmed.com). Meditation practices may impact factors such as cognition, emotions, perception, and attention, and a new field of contemplative neuroscience aims to elucidate changes in brain structure and function related to meditation (Brown and Ryan, 2003; Holzel et al., 2007; Jha et al., 2007, 2010; Srinivasan and Baijal, 2007; Luders et al., 2009; MacLean et al., 2010; Boccia et al., 2015; Vieten et al., 2018). Other researchers have begun to explore the underlying mechanisms of meditation such as impact on inflammatory modulators, cell-mediated immunity, and aging (Black and Slavich, 2016).

The term “meditation” may include a variety of practices and the definition may be open to interpretation. For practitioners, meditation is typically a spiritual practice aiming for self-actualization, enlightenment, or transcendence. From a scientific perspective, meditation is a method for distinct attentional training to improve insight into one's own mental activity (Brandmeyer et al., 2019). Meditation, as defined from a Western perspective for studies, may broadly include mantra meditation, mindfulness training, yoga, transcendental meditation, qigong, tai chi, and guided imagery (Chen et al., 2012).

Meditation has been investigated as a treatment for anxiety and mood disorders. Although current evidence cannot support meditation as a monotherapy, a 2012 meta-analysis by Chen et al. (2012) concluded that it was effective in reducing anxiety symptoms. This is noteworthy, as more than half of Americans will likely experience anxiety or mood disorders over the course of a lifespan (Alegria et al., 2016), and many are seeking complementary and integrative treatment options such as meditation. Meditation was as effective as other alternative therapies for anxiety (exercise, music therapy, relaxation practices, etc.) (Chen et al., 2012).

Despite being recently discovered by the Western world, the practice of yoga has existed for thousands of years. In the Yoga Sutras, a comprehensive set of ancient texts about yoga written by Patanjali, there are eight limbs of yoga. These include: (1) Yama (ethical standards), (2) Niyama (self-discipline), (3) Asana (postures), (4) Pranayama (breath control), (5) Pratyahara (withdrawal from senses), (6) Dharana (concentration), (7) Dhyana (contemplation), and (8) Samadhi (union). Yama and Niyama involve what not to do and what to do, respectively, in living. Asana involves physical postures and is most commonly associated with the current Western view of yoga. Pranayama involves breath control and is utilized in kriya yoga practices. Pratyahara is a process of consciously not reacting to the external world. Dharana is the process of focusing on a particular object for an extended period of time. Dhyana, or Dhyan, involves intense contemplation. Many traditions, such as Zen Buddhism, are based on this limb. The eighth limb, Samadhi, involves a sense of unification or consciousness. The combined practice of Dharana, Dhyana, and Samadhi is referred to as a process called Samyama.

The Samyama process has been around for thousands of years, although it has typically not been offered to the general population. The Isha Foundation periodically offers the advanced meditation Samyama program to people who have completed previous requirements, and it is an 8-day residential program usually held at the Isha Yoga Center in Coimbatore, Tamil Nadu, India. In 2018, this program was conducted at the Isha Institute of Inner Sciences located in McMinnville, TN, USA. Samyama is the most advanced meditation program offered by Isha Foundation for the general population, requiring a substantial number of prerequisite programs and preparation to attend. Furthermore, potential participants are evaluated before the preparation process to assess suitability to attend the program. Preparation requires about 2 months of vegan diet and daily practice of hata yoga (physical postures), kriya yoga (breathing and sound), and Shoonya meditation (conscious non-doing). No previous scientific studies have been conducted to assess the physiological and psychological impact of Samyama.

Although the impact of Samyama has not been scientifically documented, there have been several prior studies demonstrating the impact of programs and practices offered by Isha Foundation. In a recent study, our group detected increased endocannabinoid and BDNF expression after Bhava Spandana program (BSP), a 4-day meditation retreat (Sadhasivam et al., 2020). Participants from a 3-month retreat showed changes in visual attention (Braboszcz et al., 2013) and increased BDNF expression (Cahn et al., 2017). A combination of several practices was shown to improve the cardiac autonomic nervous system and increase the parasympathetic response (Muralikrishnan et al., 2012). Other studies have demonstrated physiological and psychological improvements with practice of Shambhavi Mahamudra Kriya, a breath and sound-based meditation technique (Selvaraj et al., 2008; Peterson et al., 2017).

One of the prerequisite programs for involves practicing the Shoonya meditation. This meditation is practiced in combination with Shakti Chalana Kriya, a breath and sound-based pranayama practice. Along with Vipassana and Himalayan Yoga, practitioners of Shoonya meditation showed higher gamma amplitude based on electroencephalographic (EEG) activity (Braboszcz et al., 2017) and regular practice of these meditations could lead to states of high brain entropy (Martinez Vivot et al., 2020). Daily practices of Shakti Chalana Kriya and Shoonya, and prior participation in BSP are prerequisites to enroll in the Samyama program.

The objective of this study was to evaluate physical and emotional health benefits before and after the Isha Samyama program using objective blood biomarkers, and validated psychological tools. Serial assessments through standardized surveys and blood biomarkers were performed upon enrollment in the Samyama program, after the preparation for the Samyama program, and immediately and 3 months after the Samyama retreat. Our hypothesis was that preparation for and participation in the Samyama program would improve physical health and psychological well-being in participants.

## Methods

### Subject Recruitment

The Isha Institute of Inner Sciences (McMinnville, TN) provided a list of registrants for the April 2018 Samyama Program. Invitation letters with study information, including a link to the online survey, were sent electronically to all registrants 2–3 months prior to the program. Study eligibility criteria included: Advanced meditation program participants of at least 18 years of age (and interested cohabitating spouses/partners). Study exclusion criteria were: Inability to read or comprehend the consent form; subjects with medical conditions in which a blood draw would be contraindicated (e.g., severe anemia); active use of marijuana, opioids, or related drugs; use of antibiotics or probiotic/prebiotic supplements within 60 days of enrollment; participants living outside of the country. Spouses who actively participate in meditation were excluded from the spousal control group.

Potential participants were provided a Study Information Sheet at the beginning of the online survey. They were given the following options: (1) survey-only participation (no blood sampling); (2) survey and blood sampling (requiring two blood samples prior to the Samyama program and two blood samples upon completion of the program); and (3) no participation. This study was reviewed and approved by the Institutional Review Board of the Indiana University School of Medicine (#1801728792). Participants and controls provided electronic informed consent for blood biomarker studies at the end of completing initial electronic surveys.

Control subjects were the significant others of Samyama participants, residing in the same household. They were asked to complete all surveys and were given the option of blood sampling at time-points 1, 2, and 4.

**Figure 1:**
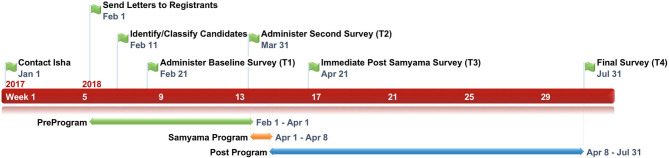
Samyama retreat timeline.

**Figure 2:**
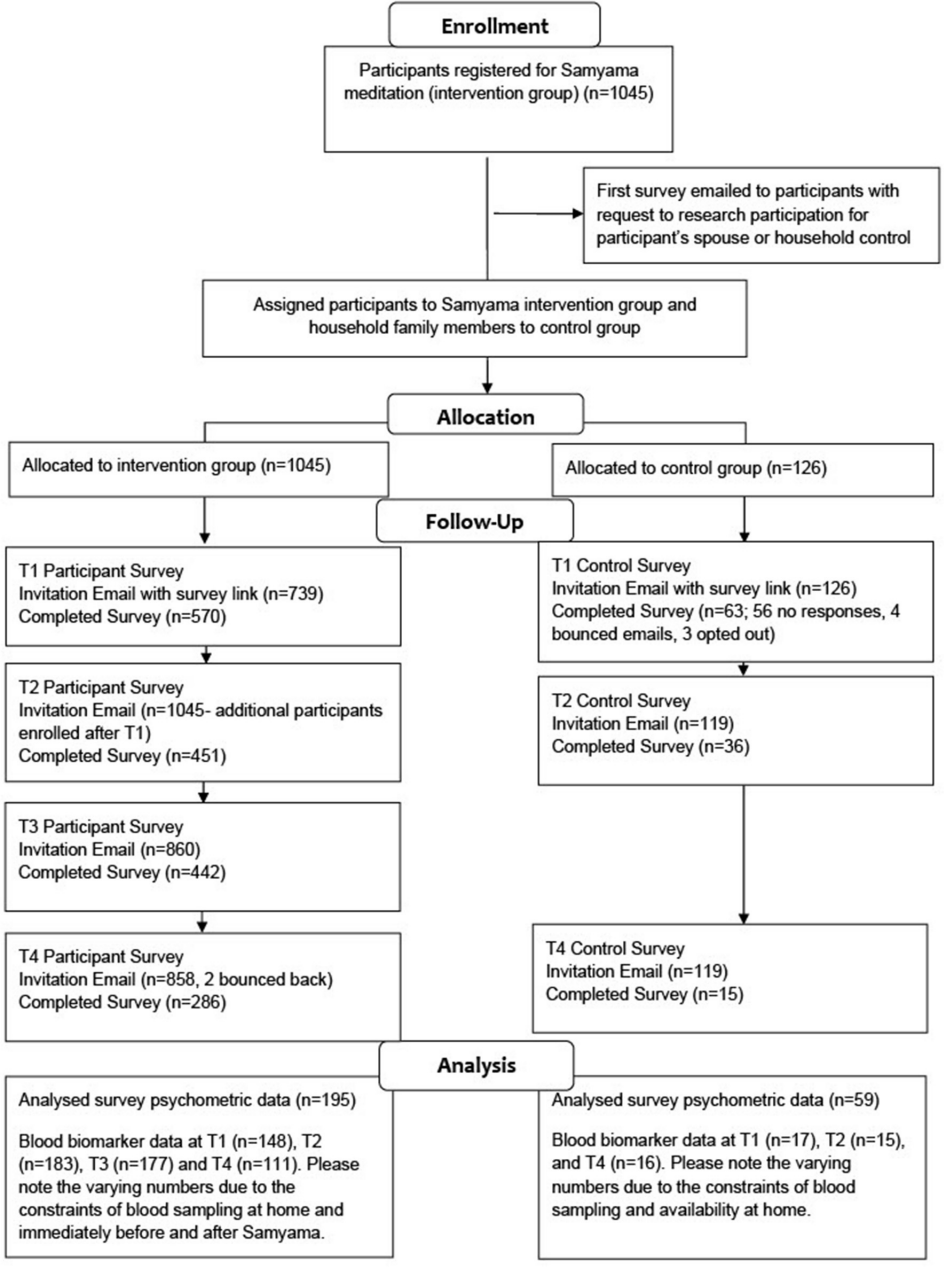
CONSORT diagram.

An overview of the study timeline is found in Figure 1, and a CONSORT (Consolidated Standards of Reporting Trials) diagram is shown in Figure 2.

### Surveys

All study participants and controls completed web-based surveys at four timepoints: (1) baseline (5–8 weeks before Samyama; T1); (2) 0–2 weeks before Samyama; T2; (3) 2–6 weeks after Samyama; T3; and (4) 3–4 months after Samyama; T4. These surveys included 10 scales that have well-established reliability and validity in the literature to assess depression, anxiety, mindfulness, joy, vitality, and resilience (Table 1). Additional questions addressed gender, race, diet, yoga practice, dietary restrictions, and overall health/well-being.

**Table 1:** Validated surveys and scoring systems.

**Characteristic**	**Survey method**	**Possible score range**	**References**
Depression	CES-D-10 Depression Scale (10 q)	0–30	Andresen et al., 1994
Emotional distress/anxiety	Emotional Distress/Anxiety Short Form (8 q)	8–40	Pilkonis et al., 2011
Mindfulness	Mindful Attention Awareness Scale (5 q)	1–6	Brown and Ryan, 2003; Osman et al., 2016
Joy	Joy subscale of the Dispositional Positive Emotion Scales (6 q)	6–42	Shioata et al., 2006
Vitality	Subjective Vitality Scale (6 q)	7–42	Bostic et al., 2000
Resilience	Brief Resilience Scale (6 q)	6–30	Smith et al., 2008

*CES, Center for Epidemiological Studies; q, questions*.

Depression was measured by the 10-item Center for Epidemiologic Studies Depression Scale (CESD-10) (Andresen et al., 1994; Hann et al., 1999). A sample item is: “During the past week, I was bothered by things that usually don't bother me.” The response is coded from 0 (rarely) to 3 (most of the time) (Radloff, 1977). The CESD-10 composite score is the sum of 10 scores.

Anxiety was measured by the eight-item Patient-Reported Outcomes Measurement Information System (PROMIS) Emotional Distress—Anxiety (Short Form) (Pilkonis et al., 2011). The scale uses a 7-day time frame, and a sample item is: “I felt fearful.” The response is coded on a 5-point scale from 1 (never) to 5 (always).

Mindfulness was measured by the five-item Mindful Attention Awareness Scale (MAAS) (Brown and Ryan, 2003; Osman et al., 2016). The MAAS is designed to assess awareness and observation of what is occurring in the present moment in participants' everyday experience. A sample statement is: “I could be experiencing some emotion and not be conscious of it until sometime later.” The response is coded on a 6-point scale from 1 (almost always) to 6 (almost never). The MAAS score is computed as the average of the five items. Many scales have been developed to measure mindfulness in recent years. Each scale addresses one or multiple aspects of mindfulness and is associated with certain strengths and weaknesses (Sauer et al., 2013). Nevertheless, MAAS remains the most widely used scale for general and clinical population in scientific research (Goh et al., 2017). Furthermore, although MAAS has been criticized for its uni-dimensionality focusing on the attentional aspect of mindfulness (Baer et al., 2006), MAAS is a more suitable scale for the nature of intensive Samyama meditation than other aspects of mindfulness, such as acceptance (Sauer et al., 2013) and non-judgmental (Baer et al., 2006) facets of mindfulness.

Joy was measured by the six-item Joy Subscale of the Dispositional Positive Emotion Scales (Shioata et al., 2006). A sample statement is: “I often feel burst of joy,” and it is coded on a 7-point Likert scale from 1 (strongly disagree) to 7 (strongly agree). The scores are computed as an average of item scores with scores ranging from 1 to 7 and higher scores indicating greater joy.

Vitality was measured by the six-item Subjective Vitality Scale (Ryan and Frederick, 1997; Bostic et al., 2000). Bostic et al. found that the short six-item scale was a better scale. A sample item is: “I feel alive and vital,” and it is coded on a 7-point scale from 1 (not at all true) to 7 (very true). The composite score is the sum of the six items, ranging from 7 to 42.

Resilience was assessed by the six-item Brief Resilience Scale (BRS) (Smith et al., 2008). A sample item is: “I tend to bounce back quickly after hard times.” The response is coded from 1 (strongly disagree) to 5 (strongly agree). The score is the average score of the six items.

### Blood Sampling

Study participants who elected blood analysis had ~10 mL peripheral blood taken at four timepoints as described above. At T1 and T4, blood draws were performed at home by an in-home phlebotomist or at an Isha Center group meditation by study personnel. The remaining samples were collected at the Isha Institute of Inner Sciences (IIIS) before/after the Samyama. All samples were collected using sterile venipuncture technique by physicians, nurses, or phlebotomists. Participants who did not submit baseline blood samples were allowed to participate at T2 and T3 (but not T4). Blood samples were de-identified and labeled with unique codes when shared with sources outside of the primary research team. Blood samples were used to assess hemoglobin A1c (HbA1c), hemoglobin (Hb), lipid profile [cholesterol, high density lipoprotein (HDL), low density lipoprotein (LDL), and triglycerides (TG)], and C-reactive protein (CRP). The study controls only had blood draws at T1, T2, and T4, as they did not participate in the Samyama program and the interval between T2 and T3 was not expected to not make significant difference. All blood draws for controls were done at home by an in-home phlebotomist and samples were sent to Indiana University.

### Sample Transfers, Storage, and Analysis

All blood samples were centrifuged, labeled upon collection by the phlebotomist, and transferred on dry ice to Indiana University. Samples were stored frozen at −80°C until analysis was performed.

### Samyama Participant Recruitment

Those who were interested in attending the Samyama program filled out an application with Isha Foundation. Each applicant was then individually assessed by an Isha Foundation instructor for suitability to attend the program. The requirement for participation in the Samyama retreat included prior completion of four Isha programs (Inner Engineering, Bhava Spandana Program, Shoonya Meditation, and Yogasanas) and a commitment to continue preparatory practices 2 months before the Samyama retreat.

### Samyama Participant Dietary Requirements

As part of the Samyama preparatory process (60 days before the program), participants were required to follow a vegan diet with at least 50% raw foods consumed. They were encouraged to avoid foods which may be considered “negative pranic,” or negative to life energy, including garlic, onion, chili, eggplant, asafoetida, coffee, and tea. Additionally, use of alcohol, cigarettes, stimulants, and illicit drugs was discouraged.

### Samyama Participant Practice Requirements

Participants were asked to perform the following practices daily for the 60-day preparation period. These include kriya yoga practices (Shakti Chalana Kriya and Shambhavi Mahamudra Kriya), hata yoga (Surya Kriya and Yogasanas), Shoonya meditation twice a day, Sukha Kriya and Arda Siddhasana for at least 1 h per day. Kriya yoga practices are combinations of posture, breath, and sound that are meant to purify and enhance the flow of one's energies while simultaneously increasing general stability. Hata yoga practices consist of postures, meant to improve flexibility and strengthen the body. Shoonya meditation is a process of conscious non-doing meant to bring stillness and stimulate the release of physical, mental, and emotional blocks. Sukha Kriya consists of alternate nostril breathing which leads to regulation of breath, meant to facilitate overall stability, balance, and steady attention. Ardha Siddhasana is a posture in which one sits cross-legged with the heel of the left foot placed at the foundational junction of one's energy channels, meant to help participants sit in stillness with spine erect for longer durations.

### Samyama Program

During the program, participants were to remain silent for the entire 8-day duration of the program. The program hall was closed to external influences. No specific instructions or programs were given to the controls, and controls did not practice any meditation.

### Statistical Analysis

Statistical calculations were performed with IBM SPSS version 25.0 (IBM Corporation, Amonk, NY) or SAS 9.4 (SAS Institute Inc., Cary, NC). To be included in the analyses, a participant was required to have completed surveys at all four assessment timepoints. For analysis of the survey results, repeated measures ANOVAs were conducted for each dependent variable with assessment time (T1, T2, T3, and T4) as the within-subject variable. The sphericity assumption was not met and thus Greenhouse-Geisser corrections were used. Partial eta-squared (η^2^) and Cohen's *d* effect sizes are reported. Cohen (1977) provides guidelines for interpreting η^2^ (small = 0.01, medium = 0.06, large = 0.14) and *d* (small = 0.2, medium = 0.5, large = 0.8).

We performed several adjustments on the survey scales as follows. The CESD-10 scale was modified, as Item 9 (“I felt lonely”) was inadvertently omitted from the administered questionnaire; therefore, it was treated as a missing item in scoring. To assess differences in outcomes in persons with and without baseline depression scores above clinical cut-offs, depression status (yes = CESD-10 score of ≥10; no = CESD-10 score of <10) was entered as a between-subjects variable. The PROMIS Emotional Distress—Anxiety Scale was also modified, as the scores were converted to T scores (mean = 50, SD = 10) with higher scores indicating more anxiety. A score of 60 (i.e., one standard deviation above average) was used as a cut-score to indicate clinically significant levels of anxiety.

Summary statistics of baseline demographics, blood tests, and weights were calculated for both controls and participants. Wilcoxon Rank Sum tests were used to compare the baseline blood and weight measurements of the participants and controls, as well as compare the final measurements for participants who continued Samyama practices. To assess changes in blood biomarker measurements over time for the participants and the controls, mixed ANOVA models with a fixed effect of assessment time (categorical) and a random intercept for subject were used to assess each measurement; Sidak adjustment were applied for multiple comparisons for blood biomarker analyses. In order to satisfy the necessary conditions for ANOVA, a rank transformation was performed prior to analysis for each of the measurements.

## Results

### Participants and Controls

Invitation letters were sent to all Samyama program registrants and controls. Baseline surveys were submitted by 569 individuals, although some participants only partially completed the survey. Follow up survey response rate for participants was 77% (445), 76% (430), and 49% (280) at T2, T3, and T4, respectively. The 195 participants who had completed surveys at all four timepoints were used for analyses of psychological parameters (Figure 2). Baseline surveys were completed by 63 controls. Follow up survey response rate for controls was 57% (Lederer et al., 2019) and 24% (Braboszcz et al., 2013) at T2 and T4, respectively (Figure 2).

Demographics are shown in Table 2; 64% of participants reported Indian race. Characteristics were similar among study participants and controls except for vegan diet (less common in controls) and body weight (higher in controls).

**Table 2:** Baseline demographics.

**Demographic**	**Participants** ** (*n* = 195)**	**Controls** ** (*n* = 59)**	***P-*value**
Race			0.10
Asian (Far East)	9 (5%)	1 (2%)	
Asian (Indian)	125 (64%)	46 (78%)	
Asian (Middle Eastern)	1 (0.5%)	1 (2%)	
Black (African American)	2 (1%)	0	
Hispanic	6 (2%)	0	
White, non-hispanic	41 (21%)	6 (10%)	
Mixed race (Asian-white)	2 (1%)	0	
Mixed race (Other)	3 (2%)	0	
Other	4 (2%)	0	
Prefer not to answer	1 (0.5%)	0	
Missing	1 (0.5%)	5 (9%)	
Sex			0.92
Female	110 (56%)	31 (53%)	
Male	85 (44%)	24 (41%)	
Unknown	0	4 (7%)	
Vegan diet	48 (25%)	4 (7%)	**0.003**
Age (years)	42.2 ± 11.6	40.4 ± 9.8	0.30
Weight (kg)	63.0 ± 11.3	72.5 ± 14.0	** <0.001**
Previous Samyama practice	43 (22.1%)	n/a	n/a

*Bolded values are significantly different (P < 0.05)*.

### Survey Results

#### Depression

**Figure 3:**
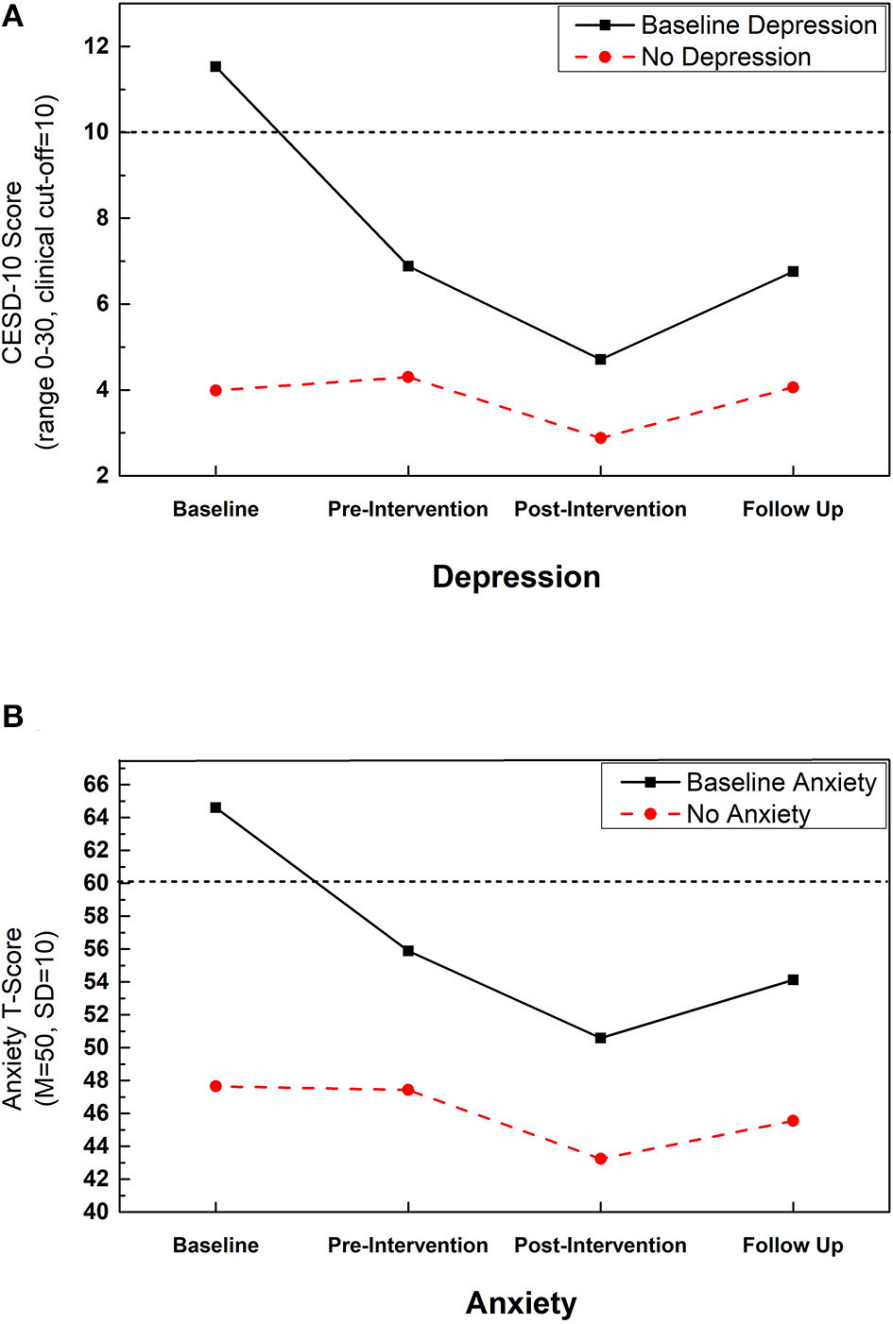
Improvements in depression and anxiety with Samyama: change in **(A)** depression and **(B)** anxiety across assessment timepoints separated by those with and without scores above the clinical cutoff at baseline for each measure. Dotted line indicates clinical cutoff for relevant measure. Depression was assessed with the 10-item Center for Epidemiological Studies Depression scale (CESD-10). Anxiety was assessed with the PROMIS Emotional Distress Anxiety—Short Form 8a.

Seventeen participants had baseline scores significant for depression (*M* = 11.53, *SD* = 2.69), with scores significantly higher (*P* < 0.01, *d* = 2.87) than in subjects not scoring above the CESD-10 cut-score (*M* = 3.99, *SD* = 2.63). For Depression (Figure 3), the main effect of time was significant [*F*_(2.64, 493.81)_ = 22.06, *P* < 0.01, η^2^ = 0.11] but was qualified by a significant interaction between time and depression status [*F*_(2.64, 493.81)_ = 14.23, *P* < 0.01, η^2^ = 0.07]. Participants without baseline depression had significantly lower scores at T3 compared with all other assessments (*P*s < 0.01, *d*s > 0.40). Participants with baseline depression had significantly higher scores at T1 compared with all other assessments (*P*s < 0.01, *d*s > 1.88), and significantly lower scores at T3 compared with all other assessments (*P*s < 0.05, *d*s > 0.53).

Overall, all participants had lower depression scores at T3 with a medium effect size (*P* < 0.01, *d* = 0.48). Persons having depression at T1 had a more precipitous drop in CESD-10 scores from T1 to T2 (large effect size; *d* = 1.88) and then to T3 (medium effect size; *d* = 0.76). The decrease in depression from T1 to T2, as well as T2 to T3, for persons without depression at T1 were small effects (*d* < 0.46). For both groups, scores at T4 had returned to T2 levels; however, for those individuals with baseline depression, T4 scores remained below the clinical cut-score for depression and did not approach baseline levels.

#### Anxiety

Fourteen participants had scores indicating baseline anxiety (*M* = 64.61, *SD* = 6.22), with scores significantly higher (*P* < 0.01, *d* = 2.28) than those without clinically significant anxiety (*M* = 47.65, *SD* = 7.52). For anxiety (Figure 3), the main effect of time was significant [*F*_(2.91, 541.91)_ = 20.23, *P* < 0.01, η^2^ = 0.10] but was qualified by a significant interaction between time and anxiety status [*F*_(2.91, 541.91)_ = 6.73, *P* < 0.01, η^2^ = 0.04]. Participants without baseline anxiety had less anxiety at T3 and T4 compared with T1 and T2 (*P*s < 0.01, *d*s > 0.22). The decrease in anxiety from T1 to T3 represented a medium effect (*d* = 0.60). Anxiety did not differ from T1 to T2 in this group (*p* = 0.72, *d* = 0.03). For participants with baseline anxiety, anxiety was significantly higher at T1 compared with all other assessments (*P*s < 0.01, *d*s > 1.25), and significantly higher at T2 compared with T3 (*P* = 0.02, *d* = 0.59). There was no significant difference in anxiety at T4 compared with T2 and T3 (*P*s > 0.14, *d*s < 0.32).

Overall, all participants showed a decrease in anxiety scores from T2 to T3 with a medium effect size (*P* < 0.01, *d* = 0.54). Persons with baseline anxiety had a more precipitous drop in anxiety symptoms from T1 to T2 (large effect, *d* = 1.26), and at T3 their anxiety scores were consistent with the mean for the normative population. Both groups evidenced a medium effect size for the decrease in anxiety from T2 to T3. For persons without baseline anxiety, anxiety remained lower at T4 compared with T2, thus suggesting some maintenance of effects over time.

#### Mindfulness

**Figure 4:**
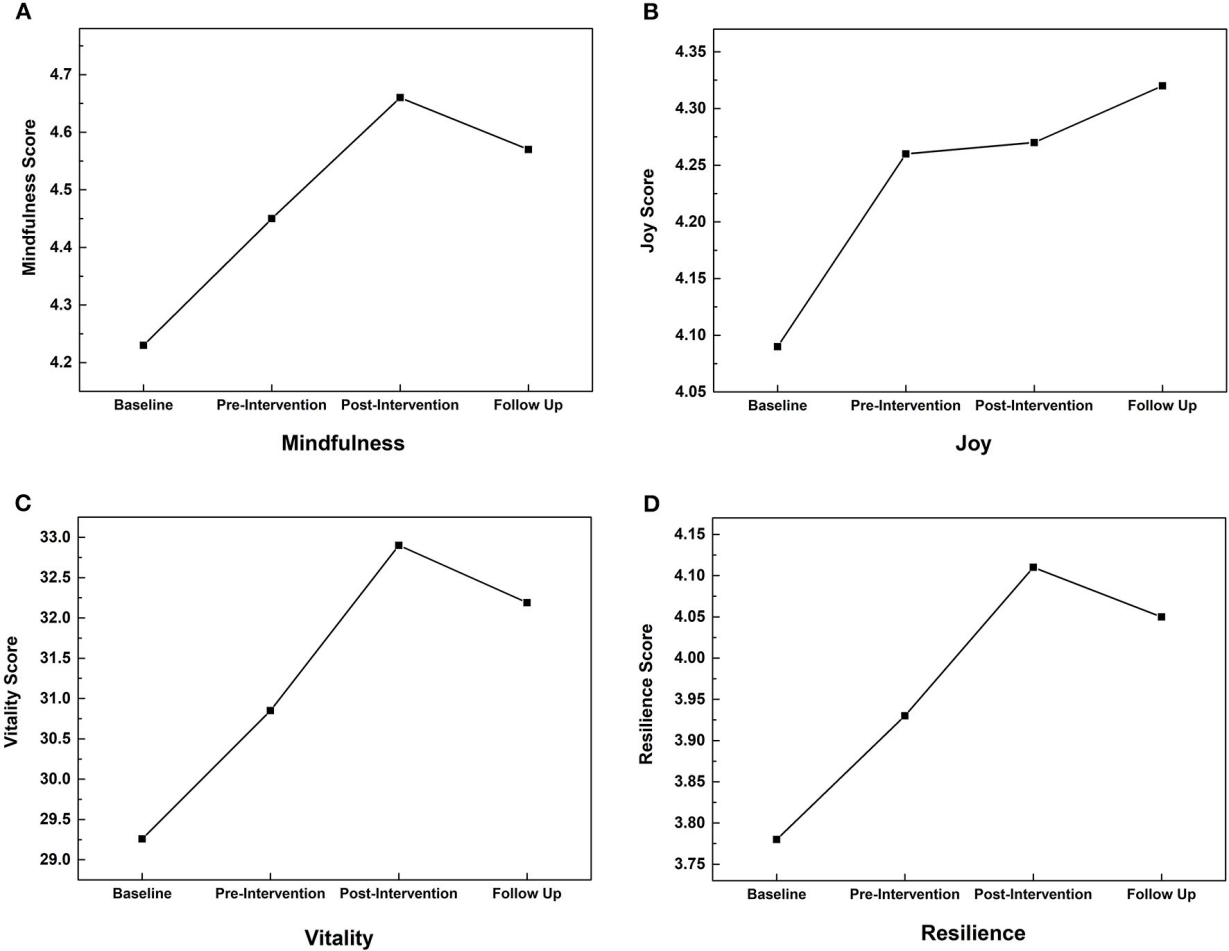
Improvements in mindfulness, joy, vitality, and resilience with Samyama: change in scores across assessment timepoints for current state of **(A)** mindfulness (State Mindful Attention Awareness Scale), **(B)** joy (Joy Subscale of the Dispositional Positive Emotion Scale), **(C)** vitality (Subjective Vitality Scale), and **(D)** resilience (The Brief Resilience Scale).

Mindfulness scores (Figure 4A) increased from T1 to T2 (*P* < 0.01, *d* = 0.23) and from T2 to T3 (*P* < 0.01, *d* = 0.24) with a significant, medium effect of time [*F*_(2.66, 516.27)_ = 18.48, *P* < 0.01, η^2^ = 0.09]. Mindfulness remained similar from T3 to T4 (*p* = 0.12, *d* = 0.10), T4 scores remained significantly higher than at T2 (*P* = 0.03, *d* = 0.13). Despite significant small to medium effects, the magnitude of change was small (T1 M = 4.23; T3 M = 4.66).

#### Joy

Joy (Figure 4B) increased from T1 to T2 (*P* < 0.01, *d* = 0.18), with a small effect of time [*F*_(2.66, 515.27)_ = 4.03, *P* = 0.01, η^2^ = 0.02]. There were no significant changes at other timepoints (*P*s > 0.49, *d*s < 0.06). Despite a significant small effect, the magnitude of change was small (baseline M = 4.09; T2 M = 4.26).

#### Vitality

There was a significant, medium effect of time [*F*_(2.77, 536.91)_ = 25.64, *P* < 0.01, η^2^ = 0.12], with vitality increasing (Figure 4C) from T1 to T2 (*p* < 0.01, *d* = 0.21) and from T2 to T3 (*P* < 0.01, *d* = 0.28). Vitality scores were similar from T3 to follow-up (*P* = 0.09, *d* = 0.10).

#### Resilience

Resilience scores (Figure 4D) increased from T1 to T2 (*P* < 0.01, *d* = 0.21) and from T2 to T3 (*P* < 0.01, *d* = 0.27) with a significant, medium effect of time [*F*_(2.88, 541.41)_ = 19.06, *P* < 0.01, η^2^ = 0.09]. There was no significant change in resilience from T3 to T4 (*P* = 0.23, *d* = 0.08).

#### Diet and Meditation Practices

Baseline data for diet and Samyama practices are found in Table 2. Beyond the baseline survey, vegan diet was reported in 90.3% (176/195) of participants and 14.6% (6/41) of controls at T2 (Table 3). Dietary surveys were not performed for T3 and T4. Survey results on active Samyama and breath watching practices are found in Table 3.

**Table 3:** Diet and meditation practices.

**Characteristic**	**Group**	**Timepoint**	**Result**
Vegan diet	Control	2	6/41 (14.6%)
	Participant		176/195 (90.3%)
Samyama practice	Participant	2	42/195 (21.5%)
		3	189/195 (96.9%)
		4	155/195 (79.5%)
Breath watching		2	n/a
		3	192/195 (98.5%)
		4	181/195 (92.8%)

In the control group, we performed similar repeated ANOVA statistical tests. We did not find any statistically significant differences in the above scores over the period of time (T1 to T4).

### Weight and Biomarker Results

#### Body Weight

Mean body weight decreased at T2 compared with T1 values (62.0 vs. 63.0 kg; *P* < 0.001). At T3, body weight was lower than all other timepoints (61.1 kg; *P* < 0.001). This represents a 3% reduction in body weight from T1 to T3. At T4, the mean body weight (62.7 kg) was similar to T1 in participants.

#### Control Group

**Figure 5:**
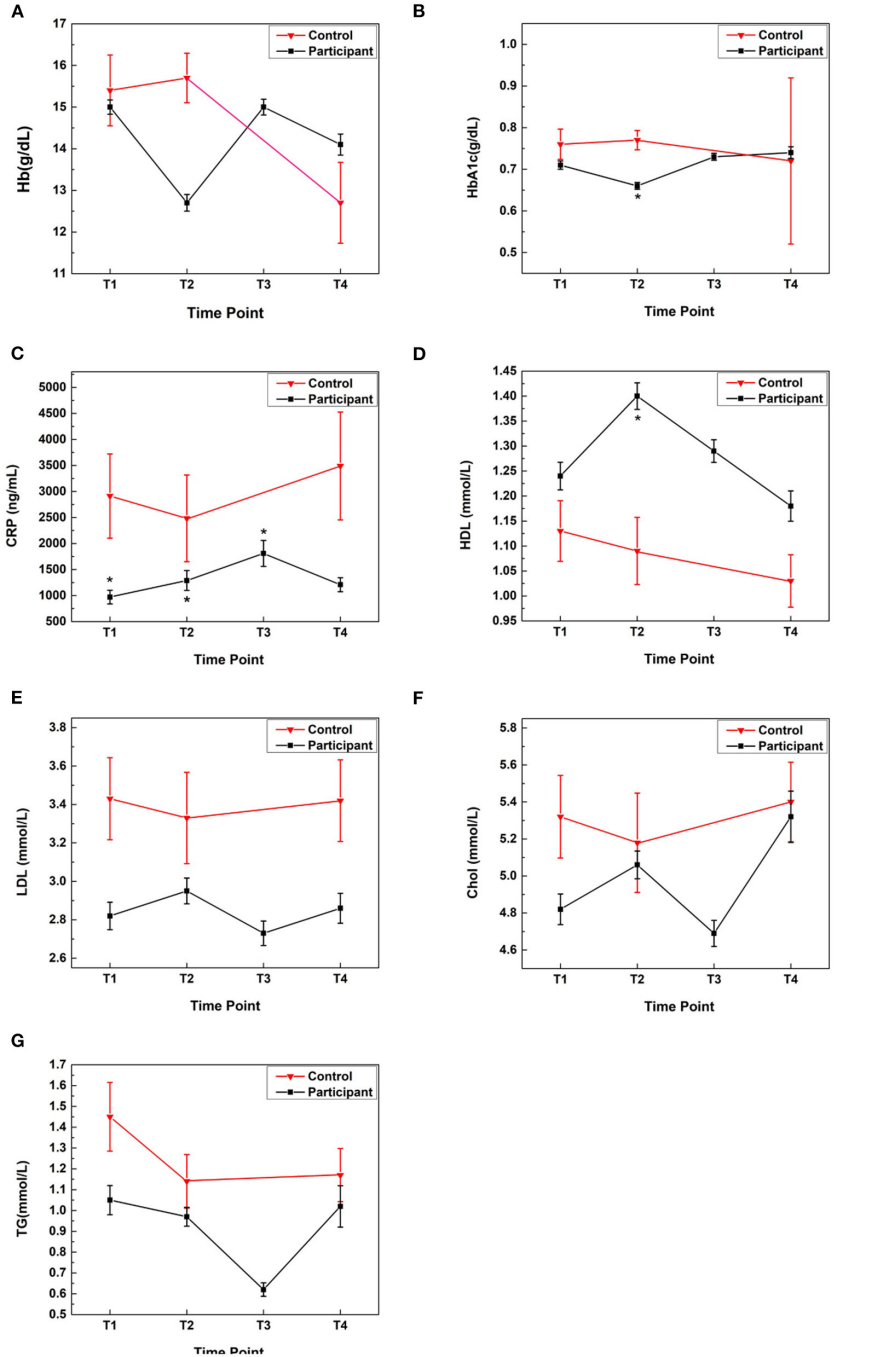
Comparisons of blood biomarkers in Samyama participants and controls: blood biomarker data was analyzed for four timepoints (T1 = baseline; T2 = immediately before retreat; T3 = immediately after retreat; T4 = 3 months after retreat). Biomarkers measured were: **(A)** Hemoglobin, **(B)** HbA1c, **(C)** CRP, **(D)** HDL, **(E)** LDL, **(F)** Cholesterol, and **(G)** Triglycerides. Compared with the controls, the participants had 2–3-fold lower CRP levels at all timepoints (adjusted *P* < 0.02). At T2, participants had significantly improved HbA1c (0.66 g/dL, adjusted *P* = 0.002) and HDL (1.4 mmol/L, adjusted *P* = 0.02) compared with controls (0.77 g/dL and 1.09 mmol/L, respectively). * Denotes statistical significance comparing participants vs. controls at a given timepoint. An adjusted *P* < 0.05 was considered significant. Chol, cholesterol; CRP, C-reactive protein; Hb, hemoglobin; HbA1c, hemoglobin A1c; HDL, high-density lipoprotein; LDL, low-density lipoprotein; TG, triglycerides.

Baseline values at T1 for the control group were higher for CRP (2,914 vs. 792 ng/mL; *P* < 0.001) and weight (72.5 vs. 63.0 kg; *P* < 0.001) compared with the participants. Controls showed no significant changes in mean CRP, HbA1C, TG, cholesterol, HDL, or LDL levels from T1 to T2 or T4. The mean Hb value for the controls decreased from T2 to T4 (*P* = 0.008) (Figure 5).

##### Participant Group Comparisons

At all timepoints, controls had 2–3-fold higher CRP levels than participants (adjusted *P* < 0.02) (Figure 5). Compared with the controls, at T2, participants had higher HDL levels (adjusted *P* = 0.02), lower HbA1c values (adjusted *P* = 0.002) and lower Hb levels (adjusted *P* < 0.001) (Figure 5).

#### Triglycerides

Participants had lower mean TG levels at T3 (0.62 ± 0.43 mmol/L) compared with T1 (1.05 ± 084 mmol/L), T2 (0.97 ± 0.61 mmol/L), and T4 (1.02 ± 1.05 mmol/L); adjusted *P* < 0.006 (Figure 5).

##### Hb

The mean Hb level in participants decreased from T1 to T2 (15.0 ± 2.1 vs. 12.7 ± 2.7 g/dL; adjusted *P* < 0.001), returned to a similar value as T1 at T3 (15.0 ± 2.5 g/dL), and decreased at T4 (14.1 ± 2.5 g/dL; adjusted *P* = 0.03). At T4, Hb levels were higher in participants than controls (14.1 ± 2.5 vs. 12.7 ± 3.5 g/dL), although the difference was not statistically significant.

## Discussion

This study adds more evidence to the growing field of meditation research to demonstrate that participation in an intense 8-day meditation retreat improved psychological and physical well-being. Depression, anxiety, mindfulness, joy, resilience, and vitality significantly improved with participation in the Isha Samyama program, and the benefits lasted for at least 3 months. Triglyceride levels and body weight also improved during the preparation and participation in Samyama—providing objective evidence of the health benefits of this program. Samyama participants had 2–3-fold lower levels of CRP throughout the study compared with household controls including at the baseline and at T4—thus indicating lower levels of systemic inflammation observed in meditators despite some of them discontinuing vegan diet at T4.

Participants maintained a strict vegan diet for 2 months during the preparation for Samyama. The vegan diet has been shown to have anti-inflammatory effects and protect against coronary artery disease (Shah et al., 2018). This diet likely influenced the biomarker results, as participants had improved HDL and HbA1C levels at T2 compared with controls. Additionally, the participants' triglyceride values decreased during this time but returned to baseline values by the end of the study. The vegan diet resulted in lower hemoglobin levels compared with controls and the baseline values at T2, but returned to the baseline and clinically normal values at T3 and T4. The vegan diet preparation is an essential and unique aspect of the advanced Samyama program. Future Samyama participants could potentially benefit from vitamin B12 and possibly iron supplements during the 2 months of preparation before the Samyama to maintain hemoglobin levels (Lederer et al., 2019). The vegan diet played a major role in the significant improvement in HbA1c and lipid profile at T2. Significantly lower CRP level observed in meditators persisted 3 months after the retreat at T4 despite most of the participants discontinuing vegan diet at T3. Furthermore, the favorable inflammatory and lipid profile parameters seen in the participants from baseline through the Samyama program is potentially due to their vegan diet, yoga and meditation practices that should protect them from cardiovascular diseases (Shah et al., 2018).

There were a few unexpected results in the blood profile. Though Samyama participants had significantly lower CRP levels at all time-points, they exhibited the higher CRP levels at T3, soon after completing the Samyama program. Samyama is an intensive process with an estimated time of meditation/yoga practice of 15 h/day over the course of 8 days. This likely resulted in acute inflammation in Samyama participants, similar to observations made in marathon runners (Takayama et al., 2018). Higher CRP levels have been linked to increased HbA1c levels (Liu et al., 2015). This could help explain why Samyama participants showed greater HbA1c levels at T3 compared to T2. Another interesting trend was that hemoglobin levels increased from T2 to T3, thought the vegan diet restrictions were still in effect. One possible explanation is that increased levels could be partly attributed to relatively less frequent hydration during the retreat.

Beneficial effects were also seen in this study regarding objective parameters such as body weight and triglycerides; however, not all of the blood biomarkers improved. CRP, HbA1C, cholesterol, HDL, and LDL levels showed no improvement over time during the study for the participants. The most likely explanation is that the study population was relatively healthy at baseline, as many were practicing Asanas, vegetarian diet, and Isha practices. The mean weight of Samyama participants was 63 kg—far below the 2015–2016 mean American weights reported by the United States Center for Disease Control and Prevention (89.7 kg for men, 77.3 kg for women) (Fryar et al., 2018). The participants likely had better baseline blood parameters compared with the average American, as they even showed better baseline body weight and CRP compared with the household controls. CRP, an indicator of systemic inflammation, was 2–3-fold lower throughout the 6-month study in the meditators compared with household controls. If more unhealthy people were to participate in the Samyama retreat, we expect that the effect on these biomarkers would be much greater.

The process of preparation for participation in the Samyama program was correlated with positive psychological outcomes. The effects on depression and anxiety are particularly noteworthy, as these conditions are increasingly common in society and non-pharmacologic interventions are being sought. For participants who reported baseline depression, the mean CESD-10 scores dropped precipitously from baseline to T2 (11.53–6.88). This effect was sustained after the retreat, as the mean score (6.76) remained well below the cutoff of 10 for clinical depression 3 months later at T4. A similar effect was observed for participants with baseline anxiety, as the PROMIS Emotional Distress-Anxiety-Short Form 8a score remained below the cutoff of 60 on all surveys beyond the baseline.

It is important to emphasize that improvements in anxiety and depression happened during the preparation phase, from T1 to T2. During the preparation phase, participants engaged in daily practice of hata yoga (physical postures), kriya yoga (breathing and sound), and Shoonya meditation (conscious non-doing). It is likely that committed daily practice of these techniques improved the conditions of those suffering from anxiety and depression.

In addition to reduction in reported anxiety and depression values, Samyama participants expressed improvements in joy, vitality, resilience, and mindfulness. All of these metrics increased from T1 to T3. Furthermore, reported joy was at the maximum value at T4 compared with all other timepoints. The other three metrics reduced slightly at T4 compared with T3, but were still considerably greater than values reported at T1 and T2. This indicates participants sustained positive psychological impacts from participating in the Samyama program.

Prior research has demonstrated the impact of yoga/meditation practices on improving stress, anxiety, and depression (Saeed et al., 2010, 2019; Wielgosz et al., 2019). Daily practice of Shambhavi Mahamudra Kriya, one of the techniques practiced during the Samyama preparation phase, resulted in reduced perceived stress and increased general well-being (Peterson et al., 2017). A one-time meditation session resulted in lower perceived stress amongst operating room professionals (Rangasamy et al., 2019). Regular practice of Sudarshan Kriya has been attributed to decreased anxiety and depression (Doria et al., 2015; Sharma et al., 2017; Hamilton-West et al., 2019; Shiju et al., 2019). Yoga practice has also been shown to improve depression in pregnant women (Gong et al., 2015; Eustis et al., 2019). For college students, taking a meditation course resulted in improvements in mindfulness, happiness, and perceived anxiety (Lemay et al., 2019; Crowley et al., 2020). Taken together, these studies demonstrate that a wide range of meditative practices can improve anxiety, depression, happiness, mindfulness, and other psychological factors.

This study did have some limitations. We could not randomize Samyama participants, as all interventions (2 months of intense practices, vegan diet, and 8 days of Samyama meditation program) are required aspects of the Samyama program. To match the controls as closely as possible for socio-economic factors, we included the Samyama participants' household controls who were not meditators and were not required to follow a vegan diet. Another limitation is the relatively smaller size of the control group compared with the Samyama participant group. Blood samples could not be collected from all participants and controls that completed surveys at all-timepoints due to logistical issues, and the blood sampling was optional. Although we followed the participants and controls for almost 5–6 months in this prospective longitudinal study, the last follow up was 3–4 months after the Samyama program. This study was not designed to assess longer-term benefits of Samyama program. Finally, there was a limited number of household controls available for this study and participant numbers decreased over the 6-month study timeframe (especially at T4).

Strengths of this study include a relatively large study population of meditators, prospective and longitudinal follow-up for 5–6 months, serial objective blood biomarkers measurements, use of multiple validated wide-spectrum psychometric tools and inclusion of a household control group accounting for matching socioeconomic status. Although the participation rate at T4 was low, a total of 195 Samyama participants still completed surveys at all four timepoints over 6 months, making this one of the largest prospective studies of advanced meditation practices. The effect size in reduction of anxiety and depression over time was one of the highest report for a meditation program. The study also assessed objective health measures including body weight and robust blood biomarkers, rather than simply using psychometric surveys, showing objective differences in meditators over time and lower systemic inflammation compared with the household controls. This has not been reported in the literature before, and these results provide objective evidence of Samyama effects on physical health and psychological well-being.

Samyama program participants suffering from pre-existing anxiety and depression benefited during the preparation phase. Therefore, further investigation is needed into these practices as possible interventions for those suffering from clinical anxiety and/or depression to determine the utility as an adjunctive therapy. Additionally, it would be worthwhile to investigate the effect of these practices on biomarker expression in a less healthy population, and potential benefits in reducing longer-term cardiovascular and diabetic risks.

## Conclusion

This study assessed two components of participation in the Isha Samyama program (1) the preparation phase with daily kriya, asana and meditation practices along with vegan diet, and (2) participation in 8-days of silent advanced Samyama meditation program retreat. Preparation and participation in the advanced Isha Samyama meditation retreat cumulatively led to significantly lower anxiety and depression as well as increased mindfulness, joy, vitality, and resilience. These improvements were sustained for at least 3 months after the retreat. Additional important and objective health benefits such as lower systemic inflammation (2–3-fold lower CRP levels), lower HbA1C, higher HDL, and body weight were also seen in Samyama participants longitudinally at T2/T3 and as well as when compared with household controls. Two months of vegan diet, yogasanas, kriyas, and meditation preparation for Samyama meditation retreat cumulatively improved HbA1 and lipid profiles in the participants while their household controls did not see any improvements (despite sharing similar socioeconomic status over the 6-month study period). The “positive pranic” and raw vegan diet together with daily practices of yogasana, kriya, and other preparatory practices had a significant impact on important blood biomarker levels in the Samyama participants. Although the mean hemoglobin level dropped with 2 months of vegan diet in the participants, it returned to baseline at completion of the retreat and 3 months later. Preparation and participation in the Samyama retreat was psychologically and physically beneficial for a large group of individuals. A relatively small percentage of Samyama participants at the baseline had scores representing high anxiety and depression. These participants were able to reduce their depression and anxiety significantly and sustained the psychological benefits 3 months after completing Samyama retreat. Future studies are needed to evaluate the sustained longer-term physical and psychological beneficial effects of Samyama in even a larger cohort as well as in those individuals diagnosed with clinical depression and/or anxiety. These future studies involving Samyama and other advanced meditation programs would provide further scientific insights into how these programs can help enhance human potential.

## Data Availability Statement

The raw data supporting the conclusions of this article will be made available by the authors, without undue reservation.

## Ethics Statement

The studies involving human participants were reviewed and approved by Indiana University Institutional Review Board. The patients/participants provided their written informed consent to participate in this study.

## Author Contributions

SS contributed to study design, data analysis and interpretation, manuscript drafting/editing, and arranging funding for this study. SA, RM, and BS contributed to study design, conduct, data collection, and manuscript revision. AW contributed to data analysis, interpretation, and manuscript drafting/revision. MM, SH, RV, and CR contributed to study conduct, data collection, and manuscript revision. SP and SD contributed to study conduct and data collection. DP contributed to research coordination, IRB approval and communications, study conduct, data collection, and manuscript revision. PC contributed to statistical analysis. JR contributed to manuscript drafting/editing. TC contributed to survey design, data interpretation, and manuscript revision. All authors have approved the submitted version, and agreed both to be personally accountable for the author's own contributions and to ensure that questions related to the accuracy and integrity of any part of the work, even ones in which the author was not personally involved, are appropriately investigated, resolved, and the resolution documented in the literature.

## Conflict of Interest

The authors declare that the research was conducted in the absence of any commercial or financial relationships that could be construed as a potential conflict of interest.

## Abbreviations

ANOVA: analysis of variance
CES-D: Center for Epidemiological Studies depression scale
CRP: C-reactive protein
Hb: hemoglobin
HbA1c: hemoglobin A1c
HDL: high density lipoprotein
LDL: low density lipoprotein
MAAS: Mindful Attention and Awareness Scale
PROMIS: Patient-Reported Outcomes Measurement Information System
TG: triglycerides.
